# Supplementary material for: Photo-tailored heterocrystalline covalent organic framework membranes for organics separation
Source: Nat Commun. 2022 Jul 2;13:3826. doi: 10.1038/s41467-022-31361-w (PMC9250524; doi:10.1038/s41467-022-31361-w)
Supplement: Supplementary file 1 — Supplementary Information [file 41467_2022_31361_MOESM1_ESM.pdf]

Supplementary Information

Photo-tailored heterocrystalline covalent organic framework membranes for organics separation

Jinqiu Yuan[1,2], Xinda You[1,2], Niaz Ali Khan[1,2], Runlai Li[3], Runnan Zhang[1,2,4], Jianliang Shen[1,2], Li Cao[1,2], Mengying Long[1,2,5], Yanan Liu[1,2], Zijian Xu[1,2], Hong Wu[1,2,6*] and Zhongyi Jiang[1,2,4,5*]

[1]Key Laboratory for Green Chemical Technology of Ministry of Education, School of Chemical Engineering and Technology, Tianjin University, Tianjin 300072, China.

[2]Collaborative Innovation Center of Chemical Science and Engineering (Tianjin), Tianjin 300072, China.

[3]Department of Chemistry, National University of Singapore, 3 Science Drive 3, 117543, Singapore

[4]Zhejiang Institute of Tianjin University, Ningbo, Zhejiang 315201, China.

[5]Joint School of National University of Singapore and Tianjin University, International Campus of Tianjin University, Binhai New City, Fuzhou 350207, China.

[6]Tianjin Key Laboratory of Membrane Science and Desalination Technology, Tianjin University, Tianjin 300072, China.

Jinqiu Yuan and Xinda You contributed equally to this work.

*Corresponding author. E-mail: wuhong@tju.edu.cn (H.W.); E-mail: zhyjiang@tju.edu.cn (Z.Y.).

This PDF file includes:

1. Materials and Methods
2. Figures 1 to 18
3. Tables 1 to 4
4. References

1. Materials and methods

1.1 Materials and reagents

1.1.1 Materials

The substrate of track-etched modified polyethylene terephthalate (PET) membranes with a pore size of 200 nm were purchased from Taoyuan Medical Chemical Instrument factory (China). The p-type cast-mono silicon chips were purchased from Suzhou Crystal Silicon Electronic & Technology Co., Ltd (China). The ultrathin carbon-coated copper grids were purchased from Beijing XXBR Technology Co., Ltd (China).

1.1.2 Reagents

1,3,5-triformylphloroglucinol (Tp, 98%) was purchased from Yanshen Development Co., Ltd (China). 2,2′-bipyridine-5,5′-diamine (Bpy, 98%) was purchased from Tensus Biotechnology Development Co., Ltd (China). Other chemicals, including p-Toluenesulfonic acid (PTSA, 98%), various solvents and dyes were purchased from Aladdin Chemical Co., Ltd. (China). The purchased chemicals were used as received without additional purification. Deionized water purified by Millipore DI system was at 18 mΩ·cm residual specific resistance.

1.2 Characterization

1.2.1 Field emission scanning electron microscopy (FESEM)

The surface and cross-section morphology images of COMs were taken by FESEM (FEI, Apreo S LoVac microscopes). For imaging of the ultrathin and fragile COMs, free-standing COMs were transferred onto PET membrane. For cross-sectional studies, the samples were freeze-fractured in liquid nitrogen. Before SEM analysis, the samples were stuck to the conductive adhesive and coated with gold by Q150T turbo-pumped sputter coater.

1.2.2 Transmission electron microscopy (TEM)

The nanoscale structure of COMs was detected by TEM (FEI Tecnai G2 F20 microscope). Ultrathin carbon-coated copper grids were used to support the free-standing COMs for TEM characterization.

1.2.3 Fourier transform infrared spectroscopy (FTIR)

The chemical structure of COMs was detected by FTIR (BRUKER, TENSOR II) with a resolution of 5 cm^{-1} using attenuated total reflectance mode.

1.2.4 Nuclear magnetic resonance spectroscopy (NMR)

Solid-state ^{13}C cross-polarization magic angle spinning (CP-MAS) NMR spectra were performed on a Varian infinity plus 300 NMR spectrometer under 12 kHz spinning rate. The samples were dried in hot air oven at 60 °C for 12 h and diced into small pieces before NMR analysis.

1.2.5 X-ray photoelectron spectrometer (XPS)

XPS spectra were performed using a K-Alpha+ spectrometer (ThermoFisher Scientific) and an Al-Ka x-ray source under high vacuum (5×10^{-8} Pa). All binding energies were calibrated using C1s peak from the adventitious carbon at 284.80 eV.

1.2.6 X-ray diffraction (XRD)

The crystallographic structure of COMs was determined by X-ray diffractometer (Rigaku, D/max-2500), scanning from 2°to 35° (2θ) a rate of 6° min^{-1}. The samples were dried in hot air oven at 60 °C for 12 h before XRD analysis to avoid solvent disturbance.

1.2.7 Steady-state photoluminescence (PL) emission spectra

The steady-state PL emission spectra and measurements were carried out using Fluorolog-3 HORIBA (Jobin Yvon). The samples were ultrasonically dispersed in solvents for fluorescence spectral analysis. The samples were excited at 300 nm, and the emission was collected from 350 to 700 nm.

1.2.8 Contact angle

The surface wetting nature of COMs was measured by contact angle goniometer (Data-Physics OCA 15EC, China) at ambient temperature. The samples were dried in a hot air oven at 60 °C for 12 h before measurement. A drop of solvent (8 μL) was placed on the membrane surface using a micropipette. The angle between the tangent line of gas-liquid-solid and the intersection point of the solid-liquid interface was then captured by a high-speed camera and calculated by drop shape analysis software.

1.2.9 Atomic force microscope (AFM)

The mechanical properties of COMs were evaluated by the Young's modulus measurement using peak

force quantitative nanomechanical mapping mode on Dimension Icon atomic force microscope system (Bruker). The COMs were supported on a rigid and smooth silicon chip.

1.2.10 Nitrogen adsorption

The N_2 sorption isotherms of COMs were measured by specific surface area and pore analyzer (BSD-PS). Based on N_2 sorption isotherms, the surface area of COMs was calculated using Brunauer Emmett Teller (BET).

1.2.11 UV-vis spectra

The concentration of the feed and permeate dye solution were determined by UV-vis spectrophotometer (U-3010, Hitachi) with a scan rate of 2 nm s^{-1} and calculated based on Lambert-Beer law.

1.3 Separation performance measurement

The permeation and rejection of COMs were measured by homemade dead-end and cross-flow filtration cells which effective filtration are is 7.85×10^{-5} m^2. Before measurements, the DPCOMs were compacted by ethanol under 2 bars for 0.5 h to obtain steady flux. Permeance was measured at driving pressure at 1 bar. The mass of permeate solution was recorded by a balance automatically per minute, and the permeance (P, L m^{-2} h^{-1} bar^{-1}) was calculated as follows,

$$P = \frac{V}{A\Delta t\Delta P} \tag{1}$$

where V (L) is the volume of permeate; A (m^2) is the effective membrane area; Δt (h) is the permeating time; ΔP is the driving pressure.

The molecular sieving was demonstrated by separating dyes in a molecular weight range of 300-1000 Da with a similar negative charge and catenulate structure. The molecular structures, dimension estimations, and charge of dyes including methyl orange (MO), Primuline (P), amido black (AB), reactive red 24 (RR24), and Evans blue (EB) were listed in Supplementary Table 4. The dyes were dissolved in ethanol at a concentration of 50 ppm (20 ppm for EB) and in 99% (v/v) ethanol-water at a concentration higher than 50 ppm. The permeate solution was collected after 1 h and the dyes rejection (%) was calculated as follows,

$$R = \frac{C_f - C_p}{C_f} \times 100\% \tag{2}$$

where C_p and C_f (ppm) are the concentration of filtration and dye feed solution, respectively. In the mixed dye separation experiment, a mixture of 20 ppm EB and 50 ppm MO in ethanol was used as the feed solution.

2. Figures

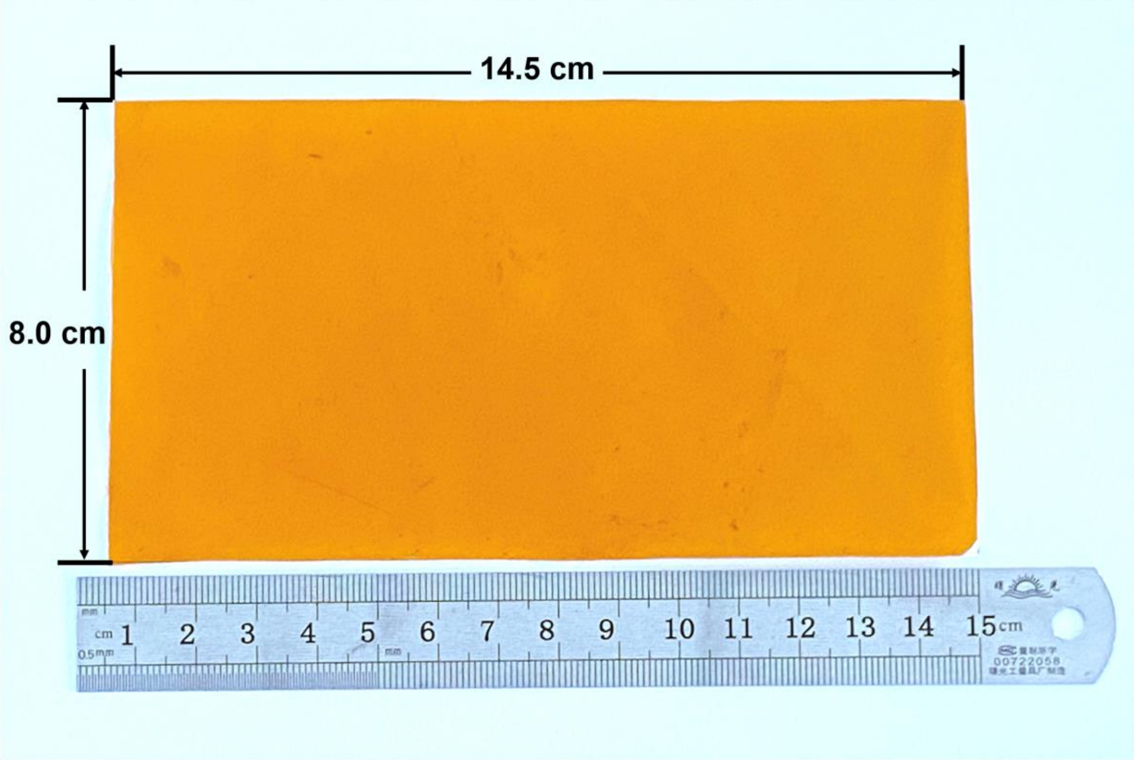

Supplementary Figure 1 The digital photograph of large-sized $DP_{2h}COM$ deposited on non-woven fabrics.

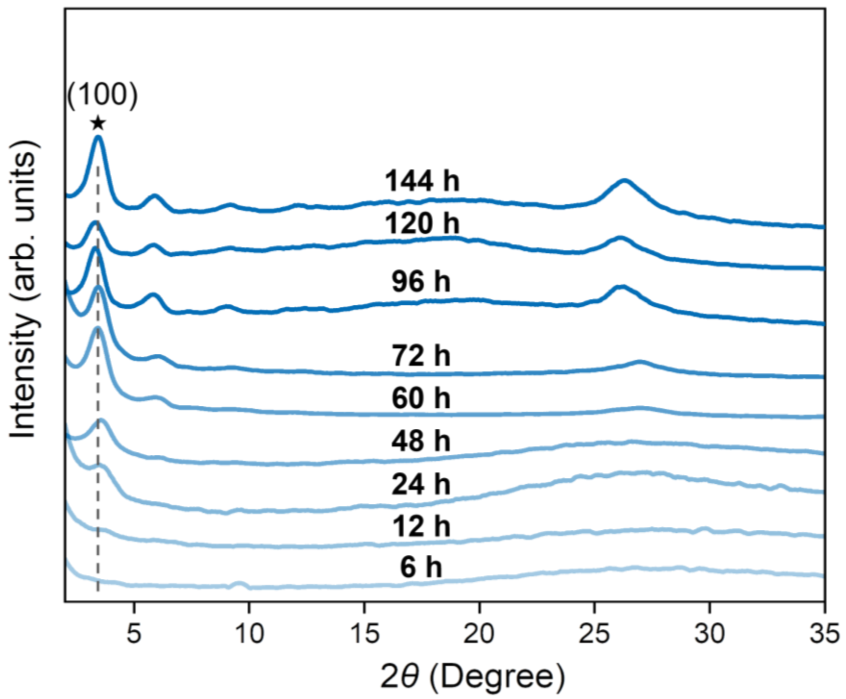

Supplementary Figure 2. XRD spectra monitoring the change in crystallinity of DCOMs with increasing fabrication time.

Note: To obtain a high-crystalline DCOM, we optimized the reaction time. The XRD patterns of DCOMs prepared at different reaction time suggest that the crystallinity of membranes increased with the reaction time and a high crystallinity could be achieved at 96 h and thereafter. Hence, reaction time of 96 h was selected as the optimum reaction time.

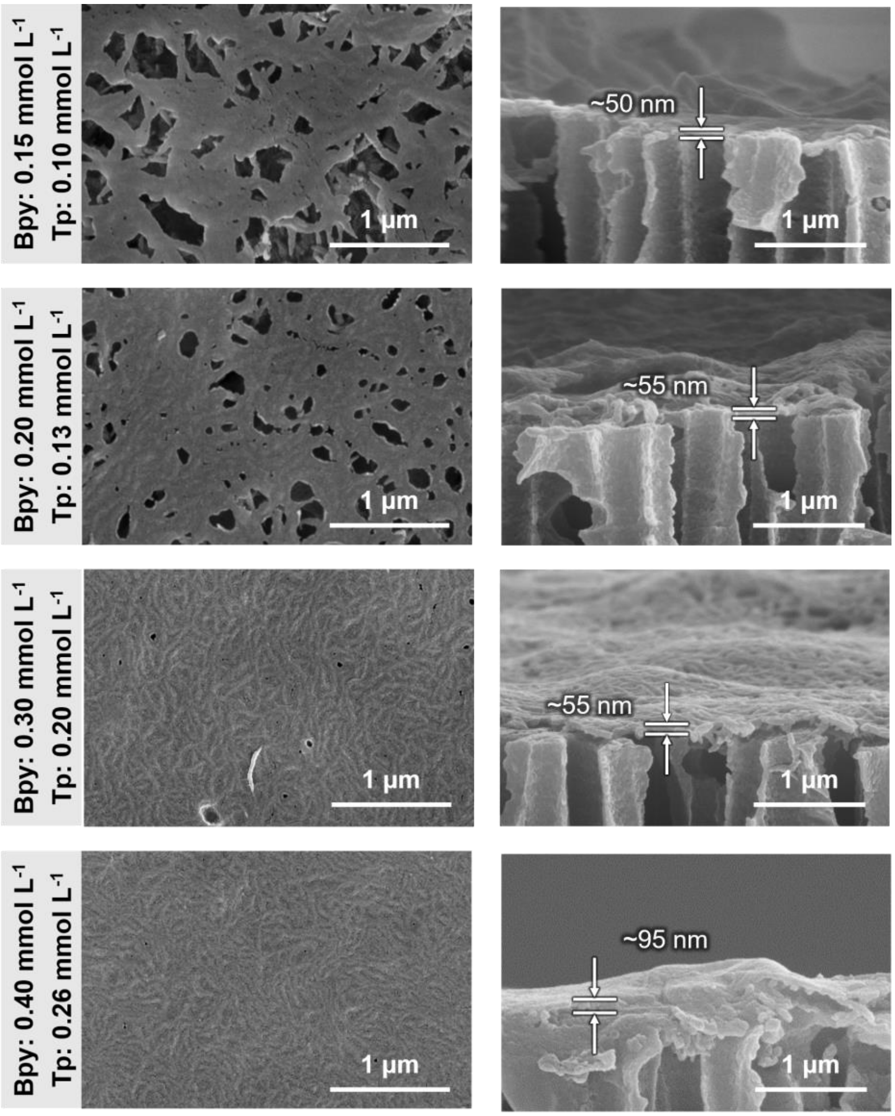

Supplementary Figure 3. Top-view (left) and cross-sectional (right) SEM images of DCOMs fabricated with varied monomer concentration. The molar ratio of Bpy (diamine) and Tp (trialdehyde) is constant at 3:2.

Note: We adjusted the monomer concentration to achieve an ultrathin DCOM. Cross-sectional SEM indicates the thickness of the DCOMs decrease to ~55 nm as the concentrations of Bpy and Tp decrease to 0.30 mmol L^{-1} and 0.20 mmol L^{-1}, respectively. Top-view SEM demonstrates the fibre-like crystal assembly morphology of high-crystalline DCOM and the inter-crystal defects are obviously observed when the membrane thickness decreased to below 100 nm. Further reducing the monomer concentration, the membrane thickness remained around 50 nm, but the inter-crystal defects became more severe.

Hence, Bpy concentration of 0.30 mmol L^{-1} and Tp concentration of 0.20 mmol L^{-1} was selected as the optimum concentration.

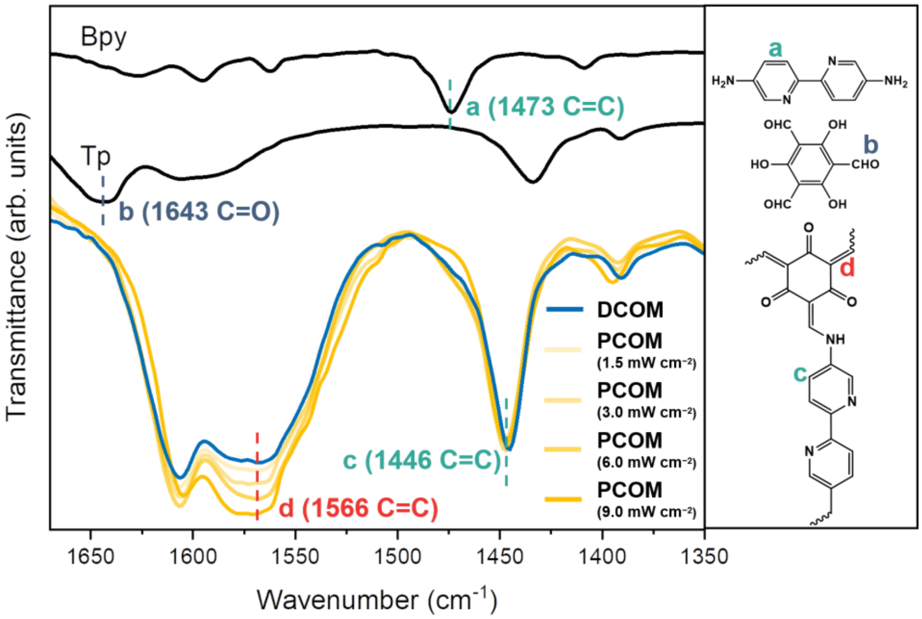

Supplementary Figure 4. FT-IR spectra monitoring the change in COMs chemical structure with increasing the irradiation intensity.

Note: With the increase of irradiation intensity, the C=C stretching band in the keto-enamine linkage of PCOMs became more intense. The increase of an irradiation intensity leads to an increased number of absorbed photons per unit time[1], thus promoting the phototautomerization of the enol-imine linkage, resulting in PCOMs with more keto-enamine linkage.

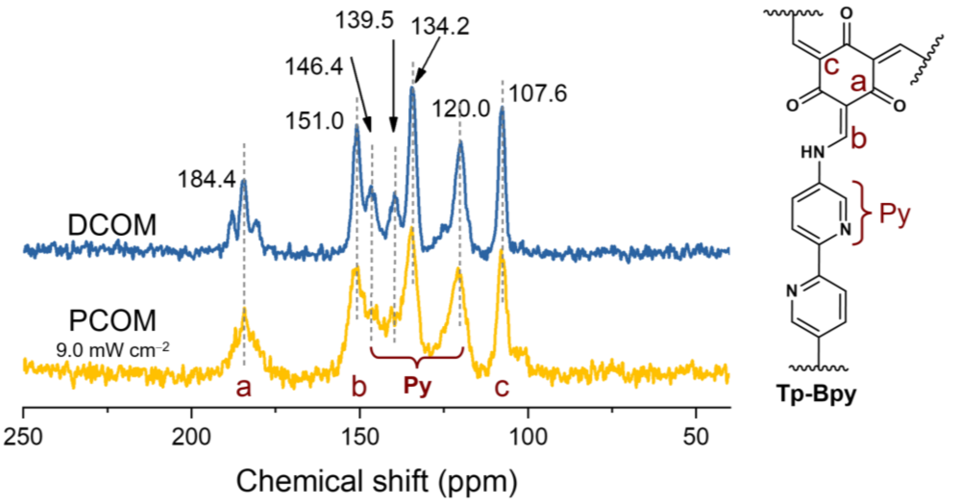

Supplementary Figure 5. ^{13}C solid-state NMR spectra of DCOM and PCOM (9.0 mW cm^{-2}). Carbon atoms responsible for the NMR resonances are labelled a-c, Py.

Note: The spectrum of DCOM exhibits resonances at 151.0 ppm and 107.6 ppm, corresponding to the enamine carbon and the α-enamine carbon, respectively, which is consistent with the reported *β*-ketoenamine-linked COF membrane[2,3]. The spectrum of PCOM shows the same resonances as DCOM, while the peaks are wider and less resolved, suggesting a lower crystalline structure[4].

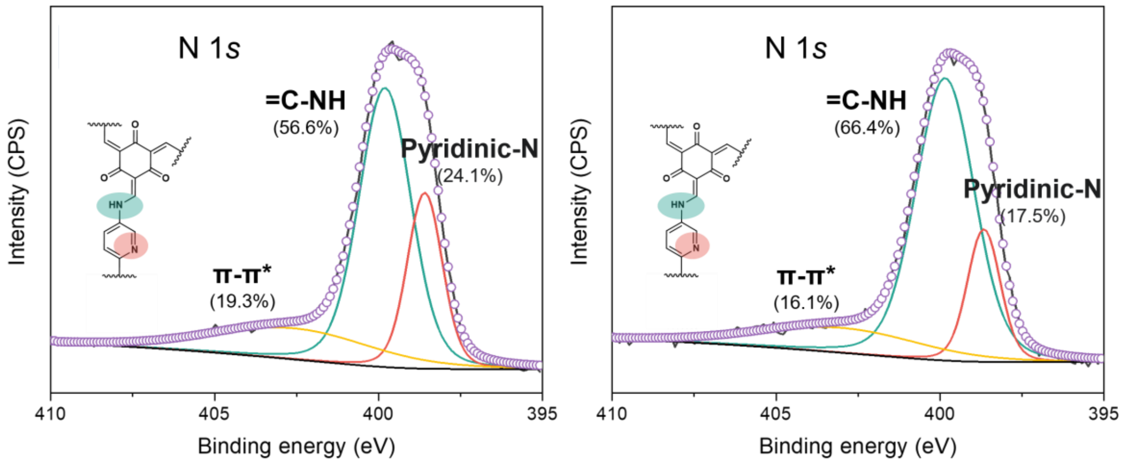

Supplementary Figure 6. High-resolution XPS spectra of the N 1*s* of (a) DCOM and (b) PCOM (9.0 mW cm^{-2}).

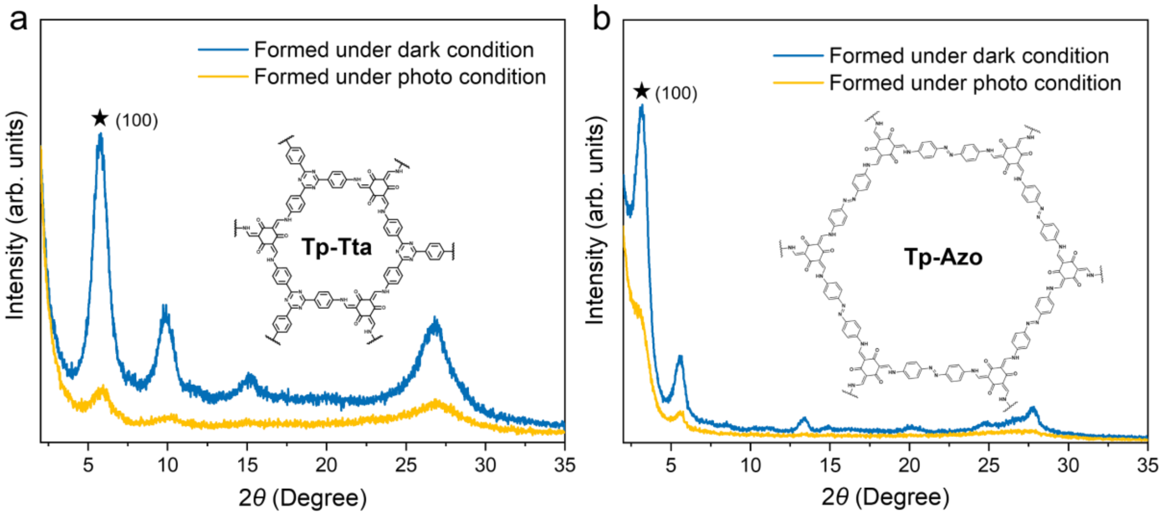

Supplementary Figure 7. (a) XRD pattens of the Tp-Tta COF membrane formed under dark condition and photo condition. (b) XRD pattens of the Tp-Azo COF membrane formed under dark condition and photo condition.

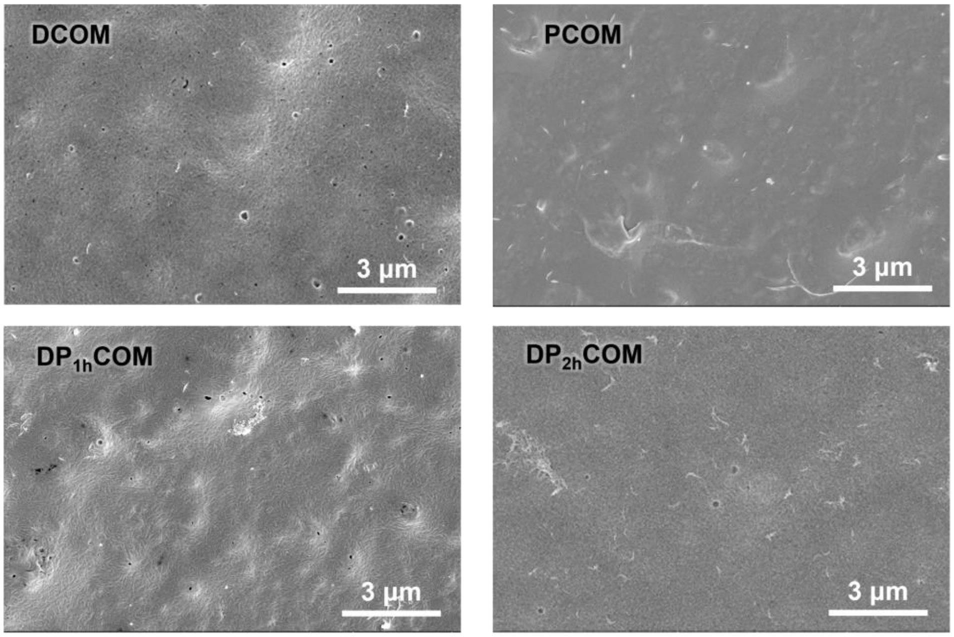

Supplementary Figure 8. Low-magnification top-view SEM images of DCOM, PCOM (9.0 mW cm^{-2}), and DPCOMs.

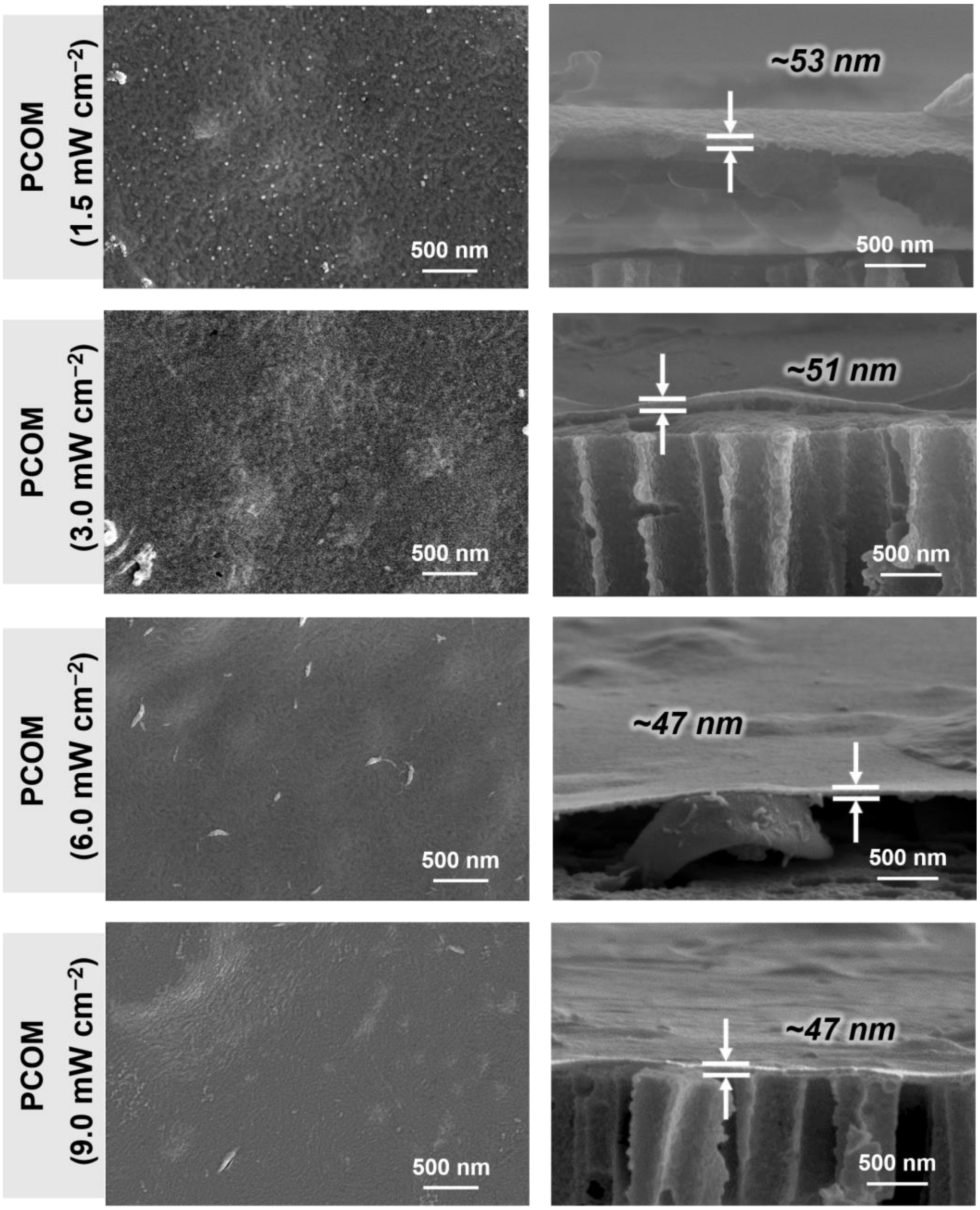

Supplementary Figure 9. Top-view (top) and cross-sectional (bottom) SEM images of PCOMs fabricated under different irradiation intensity.

Note: The defects of PCOMs gradually disappear as the irradiation intensity increases from 1.5 mW cm^{-2} to 9.0 mW cm^{-2}, suggesting that the processability of COF would improve as crystallinity decrease. Meanwhile, the thickness of membranes slightly decreases as the irradiation intensity increase. This is because, during interfacial polymerization, the defect-free membrane formed between the two immiscible phases can act as a barrier, preventing monomer diffusion and membrane growth[5].

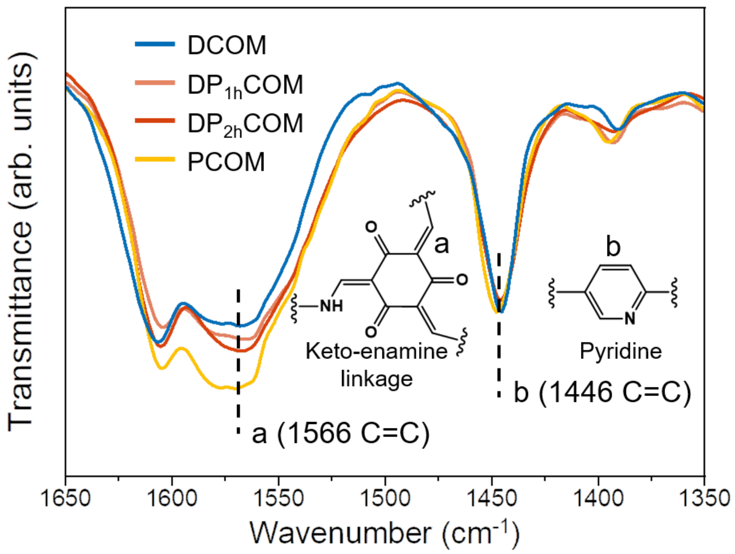

Supplementary Figure 10. FT-IR spectra of DCOM, DPCOMs, and PCOM (9.0 mW cm^{-2}).

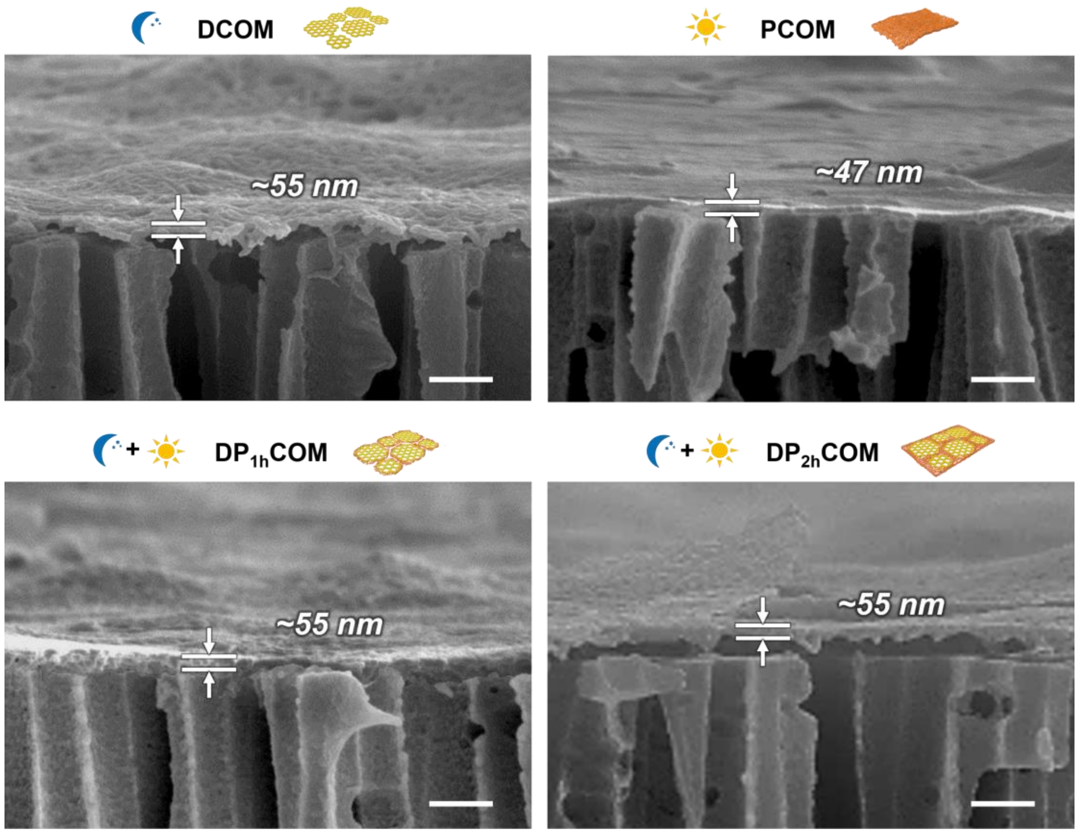

Supplementary Figure 11. The cross-sectional SEM images inset showing the thickness of DCOM, PCOM (9.0 mW cm^{-2}), and DPCOMs. Scale bar = 500 nm.

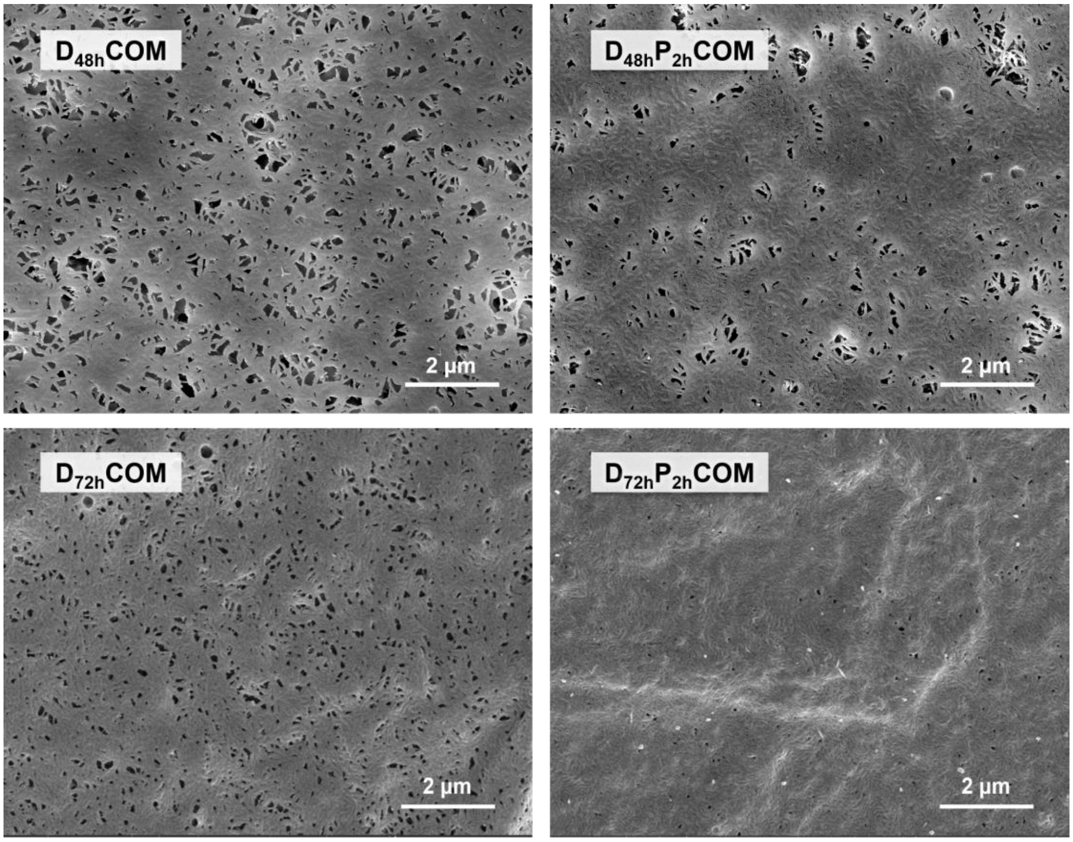

Supplementary Figure 12. Top-view SEM images of D_xCOM and $D_xP_{2h}COM$.

Note: We also varied the time of dark reaction (x) to prepare $D_xP_{2h}COM$. Compared with D_xCOM, the decreased defects of $D_xP_{2h}COM$ demonstrate the low-crystalline regions can seal the intercrystalline defects even at several hundred nanometers.

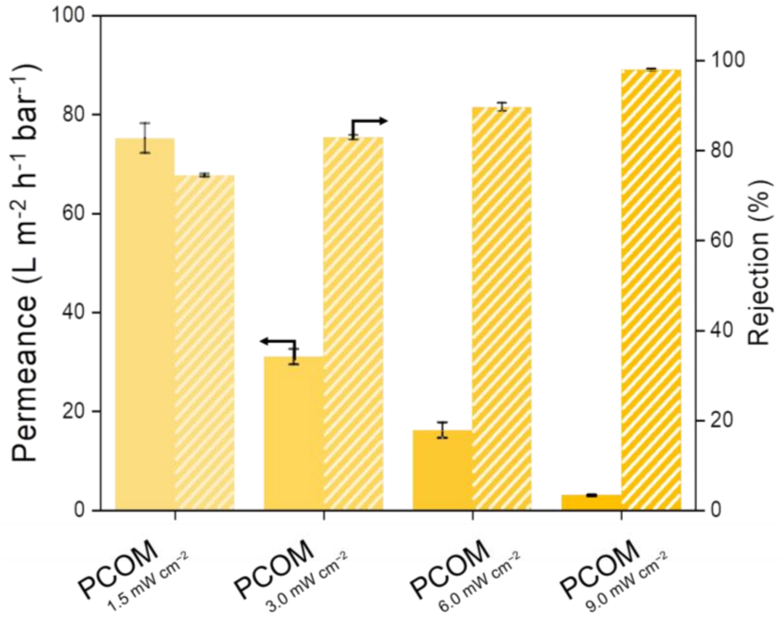

Supplementary Figure 13. Pure ethanol permeance and EB dye rejection of PCOMs prepared under varied irradiation intensity. All the error bars in this figure represent the average deviation (n = 3 independent experiments), data are presented as mean values ± SD.

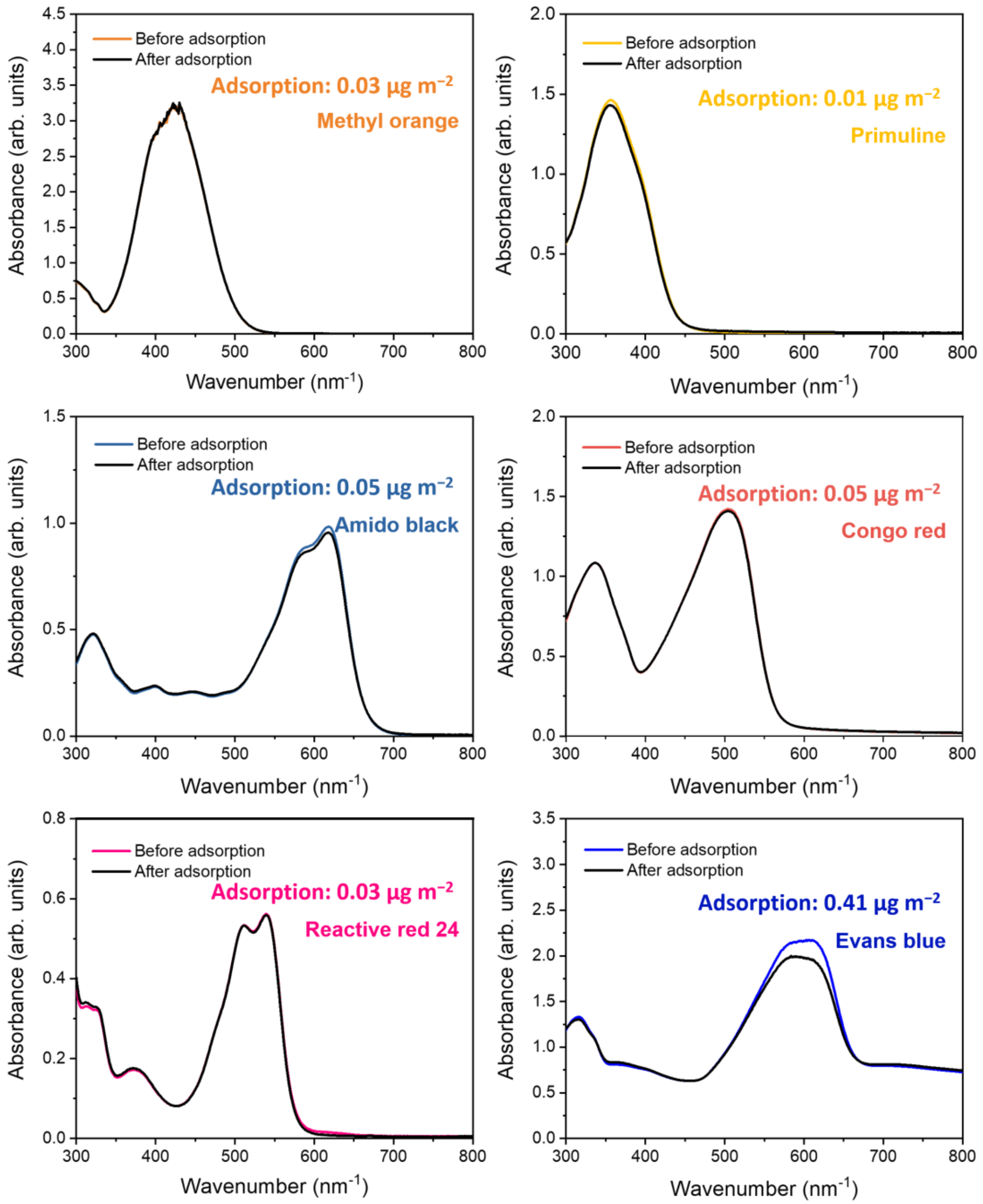

Supplementary Figure 14. UV−vis spectra of the dye solution before and after adsorption by $DP_{2h}COM$.

Note: Before measurements, all membranes were dried at room temperature prior and then placed on various dye solutions for 48 h at room temperature (25 °C). As shown in Supplementary Figure 7, the concentration of various dyes solution decreases no more than 10% after 48-h adsorption and the adsorption quantity (M, μg m^{-2}) was calculated as followed,

$$M = \frac{V}{A} \times (c_0 - c_1) \quad (3)$$

where C_0 (μg L^{-1}) is the concentration of dye solution before adsorption; C_1 (μg L^{-1}) is the concentration of solution after 48-h adsorption; V (volume of solution) = 3×10^{-3} L; A (area of membrane) = 7.85×10^{-5} m^2. The calculated adsorption quantity of the $DP_{2h}COM$ to these dyes is very small as low as 0.41 ug m^{-2}.

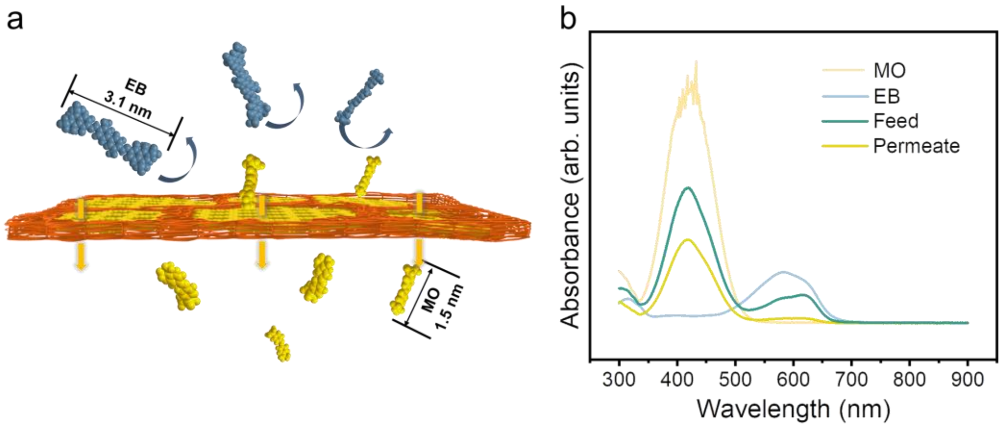

Supplementary Figure 15. **a**, The schematic diagram of $DP_{2h}COM$ showing selective molecular sieving of EB and MO. **b**, The UV-vis absorption spectra of the pure EB solution, MO solution, mixed solution before and after filtering.

Note: The mixed solution of MO (327 Da) and EB (961 Da) is added to the ultrafiltration cell with continuous stirring. After filtering, the UV absorption spectra reveal that the EB is almost completely rejected by $DP_{2h}COM$, while the MO could pass through freely.

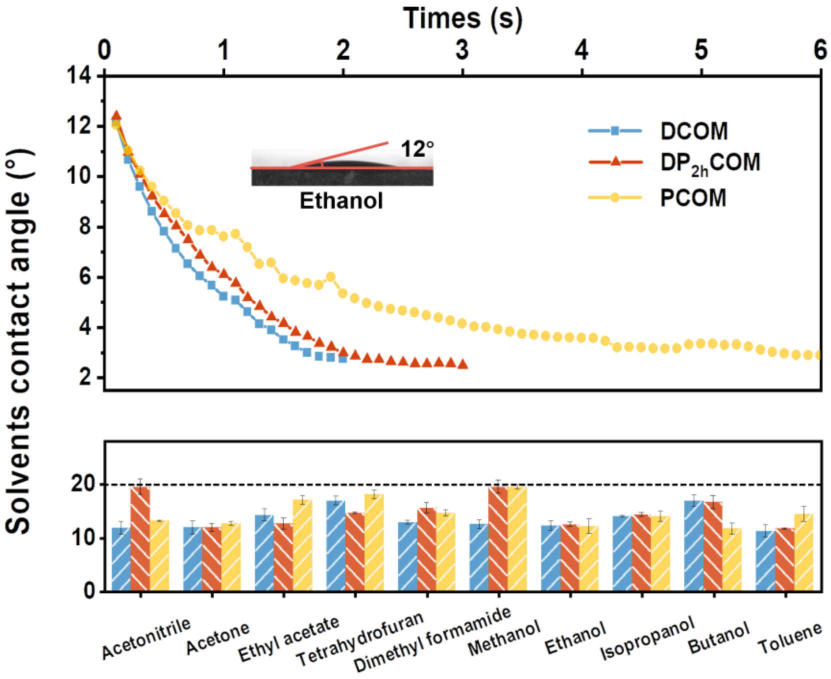

Supplementary Figure 16. Dynamic ethanol contact angle and initial solvents contact angle of organophilic COMs. The inset photograph showing the small initial ethanol contact angle of $DP_{2h}COM$. All the error bars in this figure represent the average deviation (n = 3 independent experiments), data are presented as mean values ± SD.

Note: The initial ethanol contact angle of the different COMs is similar, but the dynamic ethanol contact angle of DCOM decreases quicker than that of PCOM (9.0 mW cm^{-2}) and slower than that of DCOF. This phenomenon is attributed to the highly porous and defect-free structure of $DP_{2h}COM$.

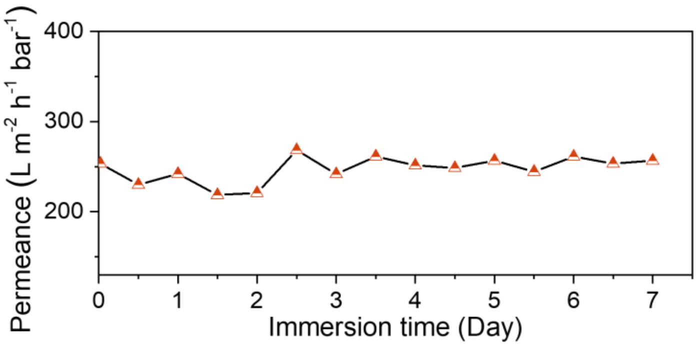

Supplementary Figure 17. Acetone permeance of $DP_{2h}COM$ after solvent immersion.

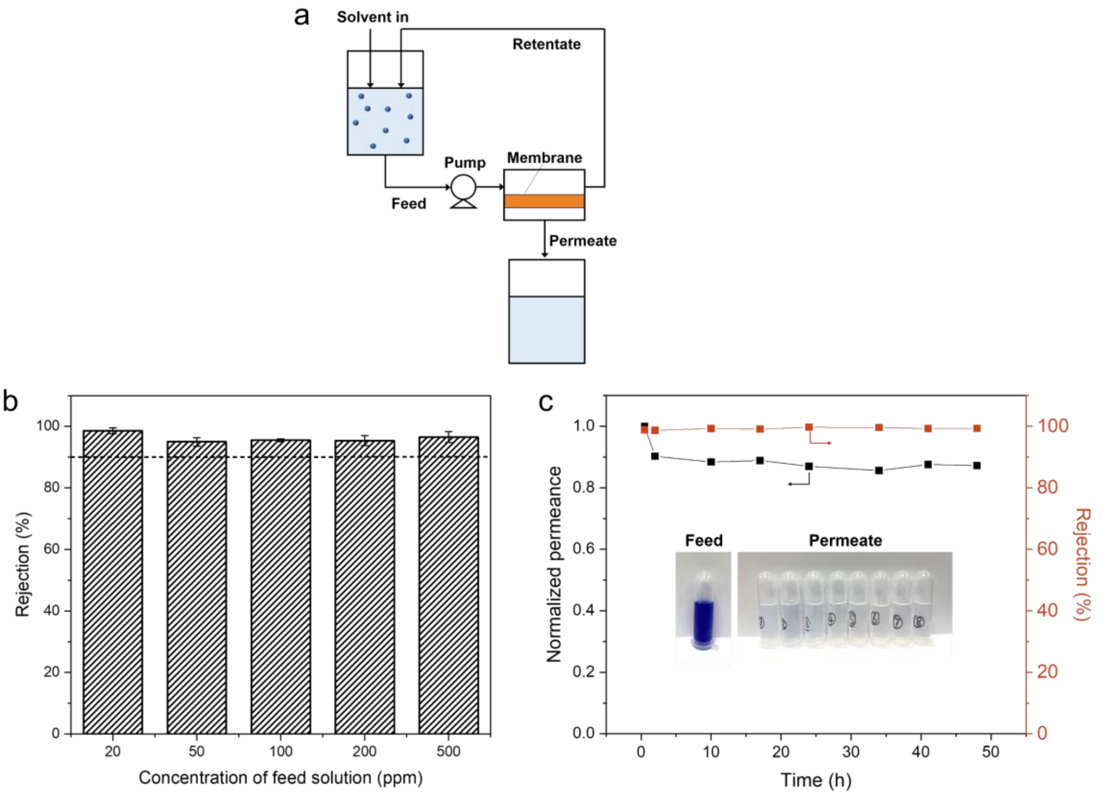

Supplementary Figure 18. a, Schematic representation of the cross-flow unit. **b,** The rejection efficiency of the $DP_{2h}COM$ for EB solution with varied concentrations. All the error bars in this figure represent the average deviation (n = 3 independent experiments), data are presented as mean values ± SD **c,** Long-term operating stability of $DP_{2h}COM$ evaluated by cross-flow unit. The inset digital photograph is the feed and permeate solution, respectively.

3. Tables

Supplementary Table 1. Comparison of the Brauner-Emmett-Teller surface area (S_{BET}), membrane thickness, pure ethanol permeance, and dye rejection of the state-of-the-art membranes

Membrane		S_{BET} (m^2 g^{-1})	Thickne-ss (nm)	Pure ethanol permeance (L m^{-2} h^{-1} bar^{-1})	Dye rejection (%)	Dye	Concentrati-on (ppm)	Ref.
Network polymers	Polyamide-CD (0.16)/PAN	-	~11	~7	~98	Brilliant blue R	10-20	[6]
	PA/ANF	-	~62	~14	~100	Erythrosin B	20	[7]
	PA/cGO/cross-linked PI	-	~15	~5	~100	Brilliant cyanin G	100	[8]
	ANF/PEI	-	~25	~3	~100	Erythrosin B	-	[9]
	PA/PAN-nanofibous-8	-	~149	~10 (methanol)	~100	Fast Green FCF	50	[10]
	β-CD-TMC	-	~115	~4	~99	Brilliant blue R	100	[11]
	TTB-CMPO/PAN	145	~50	~8	~96	Brilliant blue G	-	[12]
	p-CPM/PAN	513	~42	~11	~100	Brilliant blue G	-	[13]
	PAR-TTSBI/PI	<40	~20	~6 (methanol)	~100	Brilliant Blue	-	[14]
	PAR@mBHPF	-	~120	~14 (methanol)	~99	Methyl blue	-	[15]
COFs	TFP-DNF	172	~40	~38	~90	Direct Black	50	[16]
	TFP-DPF	336	~38	~105	~35	Direct Black	50	[16]
	trans-CON	-	~52	~10	~98	Brilliant blue R	20	[17]
	pDA/TpPa(W/E)-COF	-	~125	~85	~95	Methyl blue	100	[18]
Other materials	HLGO	-	~8	~5	~97	Brilliant blue	20	[19]
	LNCM	-	~50	~5	~99	Brilliant blue	100	[20]
This work	$DP_{2h}COM$	1443	~55	81	~95	Reactive red 24	50	
	$DP_{2h}COM$	1443	~55	264 (methanol)	~95	Reactive red 24	50	

Note: These data are estimated from the corresponding reference in which dye (700~900 Da) rejection measurements are conducted in organic solvents.

Supplementary Table 2. The molar weight, chemical structure, and charge of various dyes.

Dyes	Molar weight (Da)	Chemical structure
Methyl orange (MO)	327	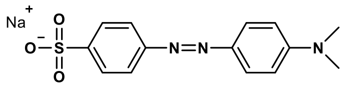
Primuline (P)	476	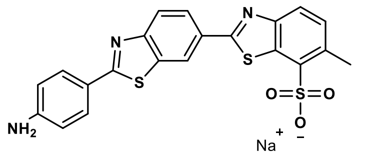
Amido black (AB)	616	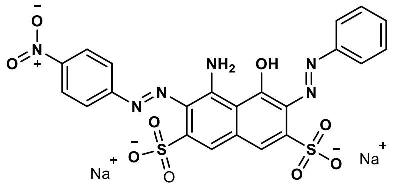
Congo red (CR)	697	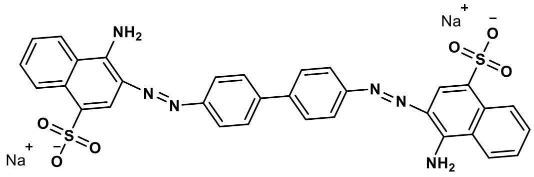
Reactive red 24 (RR24)	788	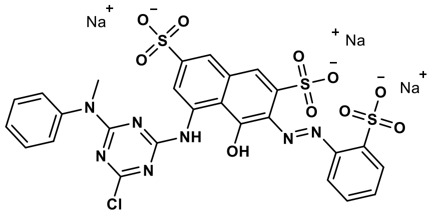
Evans blue (EB)	961	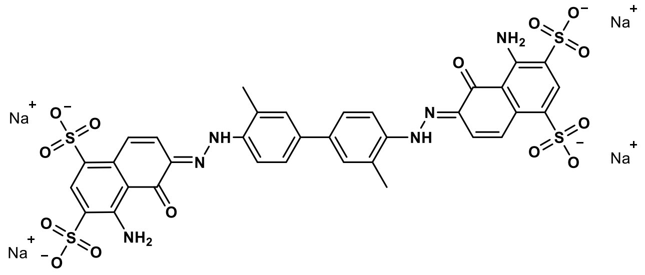

Supplementary Table 3. Dyes rejection of the state-of-the-art membranes

	Membrane	Symbol	Dye	Molecular weight (Da)	Rejection (%)	Solvent	Concentra-tion (ppm)	Ref.
Network polymers	p-CPM/PAN		Azobenzene	182	~10	Ethanol	-	[13]
			Methylene blue	320	~48			
			Fluorescein-4-Isothiocyanate	389	~64			
			Protoporphyrin IX	563	~99			
			Brilliant blue G	854	~100			
	PAR-TTSBI/PI		Brilliant blue R	826	~100	Methanol	-	[14]
			Crystal violet	408	~99			
			Disperse red	314	~92			
			Chrysoidine G	249	~77			
	TTB-CMPO/PAN		Azobenzene	182	~24	Ethanol	-	[12]
			Methylene blue	320	~65			
			Fluorescein-4-Isothiocyanate	389	~76			
			Protoporphyrin-IX	563	~97			
			Brilliant blue G	854	~96			
	PAR@mBHPF		Methylene blue	320	~22	Methanol	-	[15]
			Crystal violet	408	~36			
			Victoria blue	506	~48			
			Methyl blue	800	~98			
			Rose bengal	1018	~98			
			Alcian blue	1299	~99			
	Polyamide-CD (0.16)/PAN		Methyl red	269	~63	Methanol	10-20	[6]
			Methyl orange	327	~74			
			Orange G	452	~95			
			Protoporphyrin IX disodium salt	607	~97			
			Congo red	697	~98			
			Brilliant blue R	826	~98			
	PA/ANF		Rose bengal	1018	~100	Ethanol	20	[7]
			Erythrosin B	836	~100			
			Eosin Y	648	~100			
			Reactive Orange 16	618	~100			
			Janus green B	511	~100			
			Sudan black B	457	~100			
			Methyl orange	327	~99			
			Disperse Orange 3	242	~55			
	PA/PAN-nanofibous-8		Sudan 4	380	~99	Methanol	50	[10]
			Crystal violet	408	~96			
			Fast green FCF	809	~100			
			Reactive black	992	~100			
	β-CD-TMC		Rose bengal	1018	~99	Ethanol	100	[11]
			Brilliant blue R	826	~99			
			Remazol Brilliant blue	627	~96			
			Indigo carmine	466	~94			

Membrane		Symbol	Dye	Molecular weight (Da)	Rejection (%)	Solvent	Concentra-tion (ppm)	Ref.
			Methyl orange	327	~86			
	MPD-0.1%-10min-ACT		HNSA	246	~98	Methanol	20	[21]
			Methyl orange	327	~100			
			Naphthalene Brown	400	~100			
			Acid fuchsine	586	~100			
	MPCM		Methyl red	269	~80	Methanol	10-20	[22]
			Methyl orange	327	~75			
			Orange G	452	~97			
			Acid fuchsine	586	~98			
			Congo red	697	~99			
COFs	TFP-DPF		Chrysoidine G	249	~1	Methanol	50	[16]
			Disperse red	314	~2			
			Crystal violet	408	~6			
			Protoporphyrin IX	607	~5			
			Direct black	782	~35			
			Reactive black	992	~71			
			Vitamin B-12	1344	~96			
			Reactive green 19	1419	~99			
	TFP-DNF		Chrysoidine G	249	~2	Methanol	50	[16]
			Disperse red	314	~9			
			Crystal violet	408	~20			
			Protoporphyrin IX	607	~85			
			Direct black	782	~90			
			Reactive black	992	~94			
			Vitamin B-12	1344	~98			
			Reactive green 19	1419	~99			
	TFP-DHF		Natural red	229	~0	Methanol	50	[23]
			Safranine O	351	~5			
			Primuline	476	~6			
			Amido black	616	~12			
			Congo red	697	~78			
			Reactive black	992	~94			
			Vitamin B-12	1344	~98			
			Reactive green	1419	~99			
	trans-CON		Reactive green	1419	~98	Methanol	20	[17]
			Brilliant blue R	826	~98			
			Vitamin B-12	1344	~97			
			Rose bengal	1018	~92			
			Indigo carmine	466	~50			
			Methyl orange	327	~26			
Other materials	HLGO		Chrysoidine G	249	~97	Methanol	20	[19]
			Disperse red	314	~96			
			Methylene blue	320	~96			
			Crystal violet	408	~96			
			Rose bengal	1018	~96			
			Brilliant blue R	826	~97			
	GO–Si2		Methyl orange	327	~53	Methanol	100	[24]
			Indigo carmine	466	~75			
			Congo red	697	~96			
			Brilliant blue R	826	~99			
			Rose bengal	1018	~92			

Membrane		Symbol	Dye	Molecular weight (Da)	Rejection (%)	Solvent	Concentra-tion (ppm)	Ref.
			Chrysoidine G	249	~23			
			Methylene blue	320	~46			
			Crystal violet	408	~66			
	LNCM		Methyl orange	327	~41	Ethanol	100	[20]
			Orange G	452	~60			
			Acid fuchsin	586	~98			
			Amido black 10B	616	~98			
			Brilliant blue R	826	~99			
			Evens blue	961	~99			
This work	$DP_{2h}COM$		Methyl orange	327	~1	Ethanol	50	
			Primuline	476	~4		50	
			Amido black	616	~21		50	
			Congo red	697	~45		50	
			Reactive red 24	788	~95		50	
			Evans blue	961	~99		20	

Supplementary Table 4. Solvent viscosity of the organic solvents used in this study

Solvent	Viscosity at 25 °C* (mPa·s)
n-Hexane	0.297
Acetone	0.31
Acetonitrile	0.34
Ethyl acetate	0.426
Tetrahydrofuran	0.457
Methanol	0.54
Toluene	0.55
N,N-Dimethylformamide	0.816
Ethanol	1.08
Isopropanol	2.058
Butanol	2.63

Note: *These data are taken from reference [25].

4. References

1 Amezaga-Madrid, P., Nevarez-Moorillon, G., Orrantia-Borunda, E. & Miki-Yoshida, M. Photoinduced bactericidal activity against Pseudomonas aeruginosa by TiO2 based thin films. *FEMS Microbiol. Lett.* **211**, 183-188 (2002).

2 DeBlase, C. R. *et al.* β-Ketoenamine-linked covalent organic frameworks capable of pseudocapacitive energy storage. *J. Am. Chem. Soc* **135**, 16821-16824 (2013).

3 Wang, X. *et al.* Assembling covalent organic framework membranes with superior ion exchange capacity. *Nat. Commun.* **13**, 1020 (2022).

4 Zhang, W. *et al.* Reconstructed covalent organic frameworks. *Nature* **604**, 72-79 (2022).

5 Freger, V. & Srebnik, S. Mathematical model of charge and density distributions in interfacial polymerization of thin films. *J. Appl. Polym. Sci.* **88**, 1162-1169 (2003).

6 Huang, T., Puspasari, T., Nunes, S. P. & Peinemann, K. V. Ultrathin 2D-layered cyclodextrin membranes for high-performance organic solvent nanofiltration. *Adv. Funct. Mater.* **30**, 1906797 (2019).

7 Li, Y. *et al.* Nanofibrous hydrogel composite membranes with ultrafast transport performance for molecular separation in organic solvents. *J. Mater. Chem. A* **7**, 19269-19279 (2019).

8 Li, Y. *et al.* Graphene oxide (GO)-interlayered thin-film nanocomposite (TFN) membranes with high solvent resistance for organic solvent nanofiltration (OSN). *J. Mater. Chem. A* **7**, 13315-13330 (2019).

9 Li, Y. *et al.* A high flux organic solvent nanofiltration membrane from Kevlar aramid nanofibers with in situ incorporation of microspheres. *J. Mater. Chem. A* **6**, 22987-22997 (2018).

10 Lu, T.-D. *et al.* Electrospun nanofiber substrates that enhance polar solvent separation from organic compounds in thin-film composites. *J. Mater. Chem. A* **6**, 15047-15056 (2018).

11 Liu, J. *et al.* Precise molecular sieving architectures with Janus pathways for both polar and nonpolar molecules. *Adv. Mater.* **30**, 1705933 (2018).

12 He, X. *et al.* Controlling the selectivity of conjugated microporous polymer membrane for

efficient organic solvent nanofiltration. *Adv. Funct. Mater.* **29**, 1900134 (2019).

13 Liang, B. *et al.* Microporous membranes comprising conjugated polymers with rigid backbones enable ultrafast organic-solvent nanofiltration. *Nat. Chem.* **10**, 961-967 (2018).

14 Jimenez-Solomon, M. F. *et al.* Polymer nanofilms with enhanced microporosity by interfacial polymerization. *Nat. Mater.* **15**, 760-767 (2016).

15 Ren, D. *et al.* Microporous polyarylate membrane with nitrogen-containing heterocycles to enhance separation performance for organic solvent nanofiltration. *J. Membr. Sci.* **610**, 118295 (2020).

16 Shinde, D. B. *et al.* Pore engineering of ultrathin covalent organic framework membranes for organic solvent nanofiltration and molecular sieving. *Chem. Sci.* **11**, 5434-5440 (2020).

17 Liu, J. T. *et al.* Smart covalent organic networks (CONs) with "on-off-on" light-switchable pores for molecular separation. *Sci. Adv.* **6**, eabb3188 (2020).

18 Zhang, Y. *et al.* Molecularly soldered covalent organic frameworks for ultrafast precision sieving. *Sci. Adv.* **7,** eabe8706 (2021).

19 Yang, Q. *et al.* Ultrathin graphene-based membrane with precise molecular sieving and ultrafast solvent permeation. *Nat. Mater.* **16**, 1198-1202 (2017).

20 Wu, M. B. *et al.* Lysozyme membranes promoted by hydrophobic substrates for ultrafast and precise organic solvent nanofiltration. *Nano Letters* **20**, 8760-8767 (2020).

21 Karan, S., Jiang, Z. & Livingston, A. G. Sub-10 nm polyamide nanofilms with ultrafast solvent transport for molecular separation. *Science* **348**, 1347-1351 (2015).

22 Huang, T. *et al.* Molecularly-porous ultrathin membranes for highly selective organic solvent nanofiltration. *Nat. Commun.* **11**, 5882 (2020).

23 Shinde, D. B. *et al.* Crystalline 2D covalent organic framework membranes for high-flux organic solvent nanofiltration. *J. Am. Chem. Soc.* **140**, 14342-14349 (2018).

24 Wang, S., Mahalingam, D., Sutisna, B. & Nunes, S. P. 2D-dual-spacing channel membranes for high performance organic solvent nanofiltration. *J. Mater. Chem. A* **7**, 11673-11682 (2019).

25 Wu, X. *et al.* Elucidating ultrafast molecular permeation through well-defined 2D nanochannels of lamellar membranes. *Angew. Chem. Int. Ed.* **58**, 18524-18529 (2019).
